# HIV and hepatitis C virus infection and co-infection among trans women in San Francisco, 2020

**DOI:** 10.1371/journal.pone.0307990

**Published:** 2024-09-23

**Authors:** Izzy Chiu, Damiana Cano, Matisse Leathers, Caitlin M. Turner, Dillon Trujillo, Sofia Sicro, Sean Arayasirikul, Kelly D. Taylor, Erin C. Wilson, Willi McFarland

**Affiliations:** 1 Fielding School of Public Health, University of California Los Angeles, Los Angeles, CA, United States of America; 2 Center for Public Health Research, San Francisco Department of Public Health, San Francisco, CA, United States of America; 3 University of California Berkeley, Berkeley, CA, United States of America; 4 Department of Epidemiology and Biostatistics, University of California, San Francisco, San Francisco, CA, United States of America; 5 Department of Health, Society, & Behavior, University of California, Irvine, Irvine, CA, United States of America; Human Sciences Research Council, SOUTH AFRICA

## Abstract

**Background:**

Transgender women (hereafter “trans women”) face social marginalization, stigma, and discrimination and experience a high burden of HIV. More recently, trans women have been identified as having a high risk for hepatitis C (HCV) infection. The interaction between these two diseases and the risks for HIV/HCV co-infection among trans women are understudied.

**Objective:**

To characterize epidemiological, behavioral, and socio-structural interactions between HIV and HCV infections among trans women.

**Methods:**

This cross-sectional study examined data from a community-based survey of trans women in San Francisco recruited through respondent-driven sampling (RDS) in 2019/2020. Face-to-face interviews collected data on demographics, medical history, drug injection practices, sexual behavior, and socio-structural factors (e.g., poverty, housing insecurity, incarceration, social support). HIV and HCV antibodies were detected using oral fluid rapid tests and prior diagnosis and treatment were collected by self-report. Blood specimens were collected to confirm antibodies using ELISA. Multinomial logistic regression analysis characterized factors associated with HIV infection alone, HCV infection alone, and HIV/HCV co-infection compared to neither infection.

**Results:**

Among 201 trans women recruited, HIV prevalence was 42.3%; HCV infection by history or current seroprevalence was 28.9%; evidence for both HIV and HCV infection was present for 18.9%. Two-thirds of trans women (67.2%) had been incarcerated; 30.8% had ever injected drugs. History of injection drug use and receiving emotional support from family were factors found in common for HIV infection, HCV infection, and HIV/HCV co-infection compared to no infection. Having a sexual partner who injects drugs was associated with HIV infection alone. Not lacking care due to cost and older age were associated with co-infection. Older age was also associated with HCV infection. Of trans women with HIV infection, 91.8% had accessed HIV care, whereas only 62% with HCV had accessed some form of care.

**Conclusions:**

Our study found high levels of HIV, HCV, and HIV/HCV co-infection among trans women in San Francisco. We found common associations between HIV and HCV through injection practices and emotional support, but having a sexual partner who injects drugs was not associated with HCV infection alone or co-infection. We note a substantial gap in the treatment of HCV for trans women, including those in HIV care, that needs to be urgently addressed.

## Introduction

Transgender women (hereafter “trans women”) have a very high prevalence of HIV but have historically been underrepresented in HIV research [1]. Hepatitis C virus (HCV) is less studied than HIV among trans women, although some data suggest that trans women may be at elevated risk [2–4]. Due to gender-related stigma, discrimination, and other socio-structural barriers, trans women face barriers to medical care, both gender-related and general care [5]. These barriers have been linked to low engagement in HIV care and prevention compared to other populations in San Francisco who are at risk for or living with HIV [6, 7]. Similar barriers and risk factors linked to HIV infection may also increase their risk for HCV infection. Possible joint risk factors include injection drug use, homelessness, substance use, and incarceration [8]. There is therefore great public health need to characterize, treat, and prevent HIV/HCV co-infection among trans women.

Co-infection with HCV is common in other groups of people living with HIV, with estimates ranging from 15–30% for people living with HIV in the United States [9]. In some studies, the odds of HCV infection are six times higher in people living with HIV compared to HIV-negative people, with the highest prevalence among people who inject drugs and men who have sex with men [10, 11]. HIV and HCV share modes of transmission. HCV is primarily transmitted through parenteral exposure to infectious blood or body fluids that contain blood, especially through injection drug use. In the Centers for Disease Control and Prevention (CDC)’s 2021 HCV surveillance data, 57% of cases with HCV reported a history of injection drug use, among cases where drug use history was recorded [12]. However, much remains unknown about HIV and HCV’s interactions among trans women.

Diagnosis and treatment of HCV have improved over the last decades. HCV infection can lead to chronic inflammation of the liver, which can result in liver injury, and later fibrotic scarring and eventually liver cirrhosis [13]. HCV infection can be treated and cured through direct-acting antiviral drugs [14]. However, HCV re-infection has been found to be common among certain groups of HIV-infected individuals, such as people who inject drugs and men who have sex with men [15–17].

Trans women may be especially susceptible to co-infection with HCV and HIV, particularly if they report multiple modes of transmission common to both infections and have overlapping needle-sharing and sexual networks among their partners. To better understand the HIV/HCV co-infection burden, we present the results of an analysis of HIV and HCV serological results and behavioral data collected among trans women in San Francisco from 2019 to 2020 as part of the CDC’s first National HIV Behavioral Surveillance survey for trans women [18].

## Methods

This is a secondary analysis of cross-sectional survey data collected from the population of trans women in San Francisco, which was one of six cities in the US participating in the National HIV Behavioral Surveillance for Transgender Women (NHBST). The current analysis only includes data from San Francisco due to testing and specific questions on HCV included only in our local supplemental questionnaire. Details of the methods for the NHBST parent study were previously published by the CDC [18] and in a local analysis for San Francisco [19]. The key methods and procedures are briefly described here.

**Fig 1:**
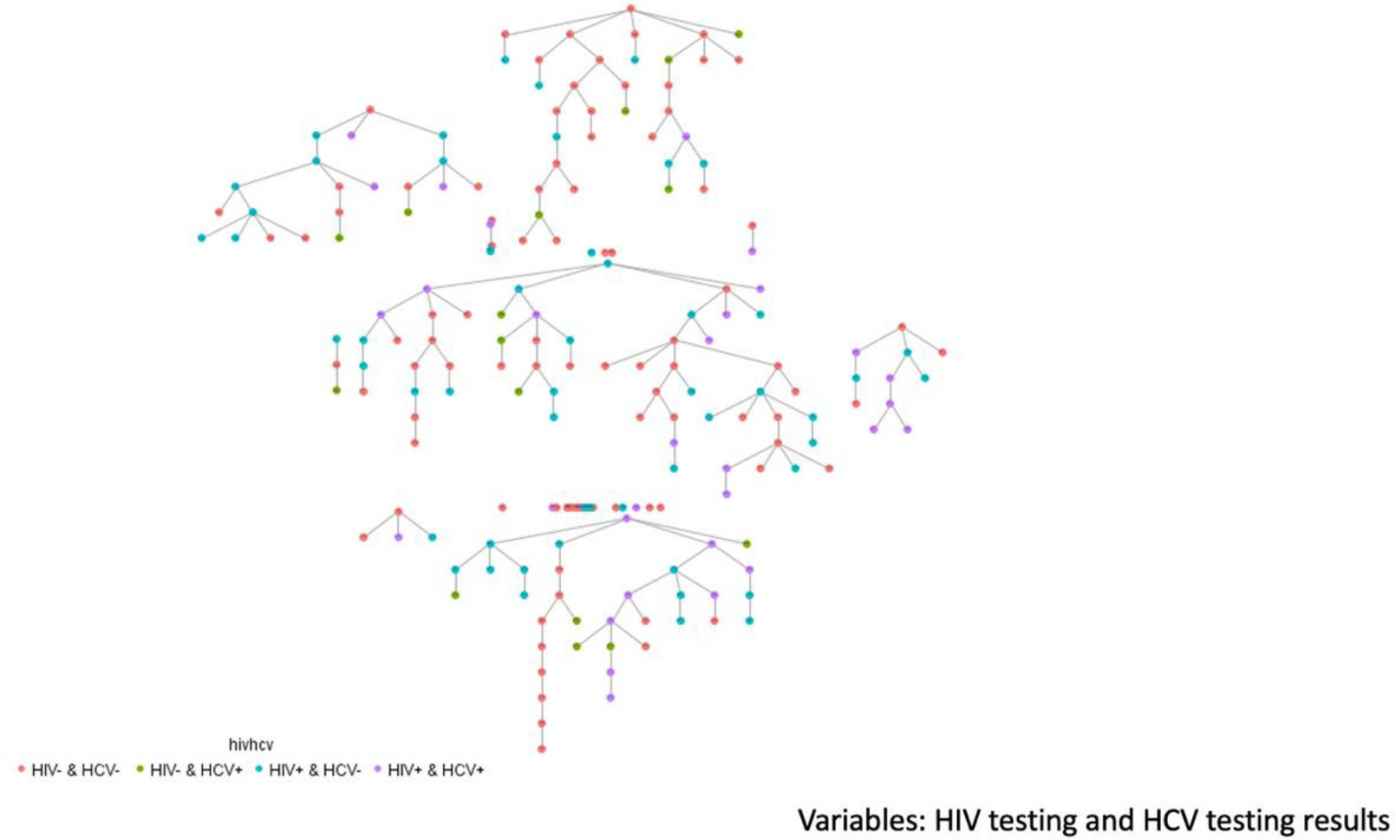
RDS recruitment tree for trans women, San Francisco, by HIV, HCV, and HIV/HCV infection status, 2020. N = 201.

Participants were recruited by respondent-driven sampling (RDS) from July 2019 to February 2020 [6, 20]. RDS starts with initial “seed” participants who are members of the trans community. Twenty-five trans women were purposively selected from persons referred by service organizations and chosen to include diverse social networks as “seeds”. Seeds included were diverse with respect to age above and below 29 years, race/ethnicity, and service organization use. Of 25 seeds, 44% produced referrals. Seeds underwent study procedures and were asked to refer up to five other eligible trans women to the survey. Eligibility criteria included self-identified trans women (i.e., currently a woman, trans woman, or other gender and had been assigned male at birth) living in San Francisco or San Mateo counties, age 18 years or older, and fluent in English or Spanish. Eligible referrals were in turn asked to refer peers to the study, and so on to create long chains of recruited participants. Recruitment continued until the targeted sample size of 200 was surpassed (final N = 201) and stability in the composition of the sample was achieved (i.e., the demographic makeup of the sample did not change with further waves of recruitment, Fig 1). The parent NHBS study sample size was based on an acceptable precision on a point estimate for HIV prevalence alone (i.e., not co-infection) [21]. Participants completing study activities were compensated $100 and were given an additional $25 for each eligible peer referral enrolled in the study.

Measures included self-reported information and HIV and HCV antibody testing results. An interviewer-administered, face-to-face questionnaire gathered information on demographic characteristics, risk behaviors (e.g., injection drug use, partner risks), socio-structural factors (e.g., homelessness, history of incarceration), health insurance status, use of medical services, and engagement in HCV care and HIV care measured as nominal variables. Ordinal variables included age group, level of education, and level of emotional support. HCV antibodies were detected using the Oraquick® HCV Rapid Antibody Test (OraSure Technologies, Bethlehem, PA, sensitivity 94.1%, specificity 99.5% [22]). Blood specimens were collected to test for HIV antibodies (Chembio Sure Check 1/2 Assay test, Chembio Diagnostics Inc, Hauppauge, NY, USA Sensitivity 99.7% Specifcity 99.9% [23]). HIV testing to confirm a positive test was done using the Oraquick® HIV Rapid Antibody Test (OraSure Technologies, Bethlehem, PA specificity of 99.9% and a sensitivity of 93% [24]). HIV viral load and prior HCV history were self-reported. Our outcomes of interest, HIV/HCV co-infection, as well as HCV and HIV infections alone, were measured based on laboratory results and medical history.

We conducted a univariate analysis of key measures including counts, proportions, and medians with interquartile ranges using STATA version 17.0 (College Station, TX). Bivariate associations of the above key measures with HIV, HCV, and HIV/HCV co-infection were assessed in cross-tabulations using the chi-square test. When a cell had an expected value of less than five, the Fisher’s Exact Test was used. We conducted a multinomial logistic regression analysis that generated three models compared to no marker nor history of either HIV or HCV infection; that is, no infections versus HIV only, versus HCV only, and versus HIV/HCV co-infection. Variables included in the multinomial logistic regression analysis were those with each infection versus not having that infection at p≤0.2 from bivariate analyses as described above, which were considered to be potential primary effects based on observed differences, findings from the literature, or potential confounders of other effects. We retained variables in the multinomial models if they were associated with the comparison to no HIV nor HCV infection at p≤0.2; those associated with infection status at p<0.05 were considered significant. There is no consensus for the use of RDS weights for bivariate and multivariate analyses, particularly for multinomial analyses [25] we therefore present unadjusted estimates. We also examined the continuum of engagement in HIV and HCV care [19, 26].

The protocol was reviewed and approved by the Internal Review Board of the University of California San Francisco (#17–24062), including a waiver for written informed consent as such a document would be the only record linking the participant to the research. All participants therefore provided verbal informed consent to preserve anonymity. Interviewers signed an information sheet to document that verbal consent was given. A copy of the information sheet was given to participants to keep if they chose.

## Results

### Participant characteristics and HIV and HCV risk-related behaviors

A total of 201 participants were enrolled with nearly two-thirds (63.7%) identifying as “trans woman”, 11.4% as “woman”, and 15.4% as another gender identity (Table 1). Most participants were trans women of color, with 37.3% Hispanic/Latina, 20.9% Black/African American, 15.9% other or multiple race/ethnicities, 7.5% Asian, and 17.9% White. A majority (59.7%) reported being homeless during the last 12 months. Nearly half (48.3%) agreed or strongly agreed that they received emotional support from their family, while 10.9% neither agreed nor disagreed. Two-thirds (67.2%) reported incarceration of over 24 hours duration in their lifetime. Most (92.5%) had health insurance and (94.5%) had ever taken hormones. One in five (19.9%) reported that they experienced difficulty accessing healthcare in the last year due to cost.

**Table 1:** Socio-demographic characteristics, familial support, healthcare access, and HIV- and HCV-related risk behaviors among trans women, San Francisco, 2019–2020.

Characteristics			n	%*
Total			201	100
Age at interview in years				
		18–24	6	3.0
		25–34	43	21.4
		35–39	21	10.4
		40–49	52	25.9
		50+	79	39.3
Gender identity				
		Woman	23	11.4
		Trans woman	128	63.7
		Woman and trans woman	19	9.5
		Other gender identity	31	15.4
Race/ethnicity				
		Black/African American, non-Hispanic/Latina/e/x	42	20.9
		Asian, non-Hispanic/Latina/e/x	15	7.5
		Hispanic/Latina/e/x	75	37.3
		White, non-Hispanic/Latina/e/x	36	17.9
		Other/Multiple	32	15.9
Current living situation				
		Own/rent	79	39.3
		Live with someone without paying rent	13	6.5
		Hotel/Single room occupancy	68	33.8
		Homeless or shelter	41	20.4
Homeless during the past 12 months			120	59.7
Education level				
		Less than high school	44	21.9
		High School or graduate equivalent	62	30.8
		Some college	69	34.3
		Bachelor’s degree or higher	25	12.4
Receives emotional support from family				
		Disagree/strongly disagree	82	40.8
		Neither agree nor disagree	22	10.9
		Agree/strongly agree	97	48.3
Below HUD [55] extremely low income			170	84.6
Ever incarcerated for more than 24 hours			135	67.2
Ever on hormones			190	94.5
Currently insured			186	92.5
Type of insurance (multiple answers allowed)				
Private health insurance			23	11.4
		Medicaid	151	75.1
		Medicare	33	16.4
		Other government insurance	7	3.5
		Veterans Administration	3	1.5
		Local public insurance	19	9.5
Unable to access healthcare due to cost, last 12 months			40	19.9
Injection drug use, ever			62	30.8
Injection drug use, last 12 months			28	13.9
Non-injection drug use, last 12 months			136	67.7
Received drugs or money in exchange for sex, last 12 months			73	36.3
Tested for sexually transmitted disease, last 12 months			117	58.2
Median sex partners, last 12 months (IQR)			3	1–6
Median exchange sex partners, last 12 months (IQR)			0	0–2.5
Sexual partner who was male having sex with men, lifetime				
		No	70	34.8
		Yes	124	61.7
		Don’t know	7	3.5
Sexual partner who had other trans women partners, lifetime				
		No	34	16.9
		Yes	158	78.6
		Don’t know	9	4.5
Sexual partner who injected drugs, lifetime				
		No	75	37.3
		Yes	115	57.2
		Don’t know	11	5.5
Sexual partner who had been incarcerated, lifetime				
		No	45	22.4
		Yes	152	75.6
		Don’t know	4	2.0
Aware of pre-exposure prophylaxis (PrEP) for HIV			187	93.0
Discussed PrEP with a provider (if not known HIV positive, n = 122)			76	37.8
Hepatitis C virus (HCV) status				
	Tested HCV positive in study, known HCV history		37	18.4
	Tested HCV positive in study, no known HCV history		11	5.5
	Tested HCV negative in study, known history of HCV		10	5.0
	Tested HCV negative in study, no known history of HCV		143	71.1
Human immunodeficiency virus (HIV) status				
	Positive		85	42.3
	Negative		116	57.7
Co-infection status				
	HIV/HCV co-infected (antibody or history)		38	18.9
	Not co-infected		173	86.1

*Categories may not add up to total due to missing data.

Table 1 also includes indicators of risk for HIV and HCV. Lifetime injection drug use was reported by 30.8%, with 13.9% injecting in the last 12 months. Over two-thirds (67.7%) used non-injection drugs during the last 12 months. Over one-third of participants (36.3%) reported receiving drugs or money in exchange for sex in the last year. Many trans women (61.7%) said they had a sexual partner who was a cisgender man who had sex with cisgender men in their lifetime, and 78.6% had a sexual partner who had other trans women partners in their lifetime. Over half (57.2%) of trans women reported having a sexual partner who had ever injected drugs. Three-fourths (75.6%) had a sexual partner who had been previously incarcerated. Most trans women (93.0%) were aware of pre-exposure prophylaxis (PrEP) for HIV.

### Bivariate analysis: Correlates of HIV, HCV, and HIV/HCV infection

Table 2 shows bivariate associations with being HIV positive (42.3%), having HCV infection by history or positive antibody test (28.9%), and having HIV/HCV co-infection by history or positive test (18.9%). Among trans women living with HIV (n = 85), 44.7% (n = 35) were positive for HCV. HIV (p = 0.036), HCV (p<0.001), and HIV/HCV co-infection (p = 0.005) prevalence increased with increasing age, with no infections detected among trans women aged 18–24 years. Black/African American trans women had the highest prevalence of HIV (66.7%), HCV (40.5%), and HIV/HCV co-infection (33.3%) among races/ethnicities, although only HIV was statistically significantly higher (p = 0.004). Trans women who agreed/strongly agreed with the statement that they received emotional support from their family were more likely to be HIV positive than those who disagreed (52.6% vs. 28.0%, p = 0.003). Trans women with health insurance had higher HIV prevalence compared to those who did not (44.6% vs 13.3%, p = 0.018). A higher prevalence of HIV infection and HIV/HCV co-infection was associated with having Medicare (48.3% vs 24.0%, p = 0.003; 33.3% vs 16.1%, p = 0.021). Additionally, HIV-positive, HCV-positive, and HIV/HCV co-infected trans women were less likely to say they were unable to access healthcare due to cost compared to trans women without these infections (all p-values<0.012). Being incarcerated for more than 24 hours was associated with a higher prevalence of HIV, HCV, and HIV/HCV co-infection (all p-values<0.003). HIV, HCV, and co-infection prevalence was higher among trans women who reported a history of injecting drugs ever (61.3%, 67.6%, 41.9%, respectively, all p-values<0.001) and in the last 12 months (64.3%, 78.6%, 57.1%, respectively, all p-values<0.011). Having a sexual partner with a history of injecting drugs was also associated with higher HIV, HCV, and co-infection prevalence (all p-values<0.001), as was having a partner who had a history of incarceration (all p-values<0.044).

**Table 2:** HIV, HCV, and co-infection prevalence by demographic and risk behaviors, trans women in San Francisco, 2019–2020 (n = 201).

				HIV positive		p-value^1^	HCV history / positive		p-value^2^	HIV/HCV co-infection by history or test		p-value^3^
				n	%^a^		n	%^a^		n	%^a^	
*Total*				85	42.3		58	28.9		38	18.9	--
*Socio-demographic characteristics*												
	Age at interview in years					**0.036**			**<0.001**			**0.005**
		18–24 years		0*	0		0*	0		0*	0	
		25–34 years		13	30.2		3*	6.98		2*	4.65	
		35–39 years		10	47.6		3*	14.3		2*	9.52	
		40–49 years		21	40.4		14	26.9		10	19.2	
		50+ years		41	51.9		38	48.1		24	30.4	
	Gender identity					0.861			0.207			0.484
		Woman		9	39.1		11	47.8		7	30.4	
		Trans woman		57	44.5		34	26.6		22	17.2	
		Woman & Trans woman		7	36.8		5	26.3		4*	21.1	
		Other gender identity		12	38.7		8	25.8		5	16.1	
	Race/ethnicity					**0.004**			0.117			0.082
		Black, non-Hispanic/Latina/o/x		28	66.7		17	40.5		14	33.3	
		Asian, non-Hispanic/Latina/o/x		6	40.0		3*	20.0		2*	13.3	
		Hispanic/Latina/o/x		26	34.7		16	21.3		10	13.3	
		White, non-Hispanic/Latina/o/x		10	27.8		13	36.1		5	13.9	
		Other/multiple, non-Hispanic/Latina/o/x		14	43.8		9	28.1		7	21.9	
	Current living situation					0.236			0.773			0.385
		Own/rent		39	49.4		26	32.9		19	24.1	
		Live with someone without paying rent		7	53.8		3*	23.1		3*	23.1	
		Hotel/SRO		24	35.3		18	26.5		11	16.2	
		Homeless or shelter		15	36.6		11	26.8		5	12.2	
	Homeless during past 12 months					0.275			0.606			0.324
		No		38	46.9		25	30.9		18	22.2	
		Yes		47	39.2		33	27.5		20	16.7	
	Education level											
		Less than high school		20	45.5	0.100	15	34.1	0.44	11	25	0.494
		High School / GED		30	48.4		21	33.9		13	21	
		Some college		30	43.5		16	23.2		11	15.9	
		Bachelor’s degree or higher		5	20		6	24		3*	12	
	Receives emotional support from family					**0.003**			0.191			0.084^4^
		(Strongly) disagree		23	28		23	28		15	18.3	
		Neither agree nor disagree		11	50		3*	13.6		1*	4.55	
		(Strongly) agree		51	52.6		32	33		22	22.7	
	Below HUD’s extremely low-income limit					0.92			0.458			0.724
		No		13	43.3		7	23.3		5	16.7	
		Yes		72	42.4		51	30		33	19.4	
*Insurance and healthcare*												
	Ever on hormones					0.682			0.211			0.95
		Yes		81	42.6		53	27.9		36	18.9	
		No		4*	36.4		5	45.5		2*	18.2	
	Ever taken unprescribed hormones (last 12 months)					0.152			0.924			0.266
		No		77	44.3		50	28.7		35	18.5	
		Yes		8	29.6		8	29.6		3*	25	
	Currently insured					**0.018**			0.431			0.208
		No		2*	13.3		3*	20		1*	6.67	
		Yes		83	44.6		55	29.6		37	19.9	
	Ever injected substances other than hormones (e.g., silicon) to change your body to match your gender identity					0.652			0. 652			0.769
		Yes		16	45.7		9	25.7		6	17.1	
		No		69	41.6		49	29.5		32	19.3	
	Insured–private health plan (through an employer or purchased directly)					0.745			0.755			0.712
		No		76	42.7		52	29.2		33	18.5	
		Yes		9	39.1		6	26.1		5	21.7	
	Insured, Medicaid					**0.003**			0.217			0.150
		No		12	24		11	22		6	12	
		Yes		73	48.3		47	31.1		32	21.2	
	Insured, Medicare											
		No		66	39.3	0.052	44	26.2	0.060	27	16.1	**0.021**
		Yes		19	57.6		14	42.4		11	33.3	
	Insured, other government plan											
		No		82	42.3	0.975	55	28.4	0.405	36	18.6	0.506
		Yes		3*	42.9		3*	42.9		2*	28.6	
	Insured, Veterans Administration											
		No		84	42.4	0.752	50	25.3	0.266	38	19.2	0.399
		Yes		1*	33.3		0*	0		0*	0	
	Insured, Healthy San Francisco Plan											
		No		76	41.8	0.638	56	30.8	0.064	37	20.3	0.110
		Yes		9	47.4		2*	10.5		1*	5.26	
	Unable to access healthcare due to cost, last 12 months					**0.005**			**0.011**			**0.012**
		No		76	47.2		53	32.9		36	22.4	
		Yes		9	22.5		5	12.5		2*	5	
*Detention and drug use*												
	Ever incarcerated for more than 24 hours											
		No		18	27.3	**0.003**	7	10.6	**<0.001**	4*	6.06	**0.001**
		Yes		67	49.6		51	37.8		34	25.2	
	Injection drug use, ever (unprescribed)					**<0.001**			**<0.001**			**<0.001**
		No		47	33.8		16	11.5		12	8.63	
		Yes		38	61.3		42	67.7		26	41.9	
	Injection drug use, last 12 months											
		No		67	38.7	**0.011**	36	20.8	**<0.001**	22	12.7	**<0.001**
		Yes		18	64.3		22	78.6		16	57.1	
	Non-injection drug use, last 12 months					0.443			0.679			0.443
		No		30	46.2		20	44.4		30	46.2	
		Yes		55	42.3		38	25		55	40.4	
*Sexual Behaviors and Partners*												
	Received drugs or money in exchange for sex (past 12 months)											
		No		57	44.5	0.394	39	30.5	0.504	26	20.3	0.5
		Yes		28	38.4		19	26		12	16.4	
	Tested for sexually transmitted disease, past 12 months											
		No		37	46.8	0.365	34	43	**0.001**	22	27.8	**0.01**
		Yes		45	38.5		22	18.8		14	12	
	Aware of pre-exposure prophylaxis (PrEP)					0.545			**0.015**			0.096
		No		7	50		8	57.1		5	35.7	
		Yes		78	41.7		50	26.7		33	17.6	
	Sexual partner who was male having sex with men, lifetime											
		No		27	38.6	0.373	22	31.4	0.554	13	18.6	0.894
		Yes		56	45.2		34	27.4		24	19.4	
	Sexual partner who had other trans women partners, lifetime											
		No		11	32.4	0.201	8	23.5	0.386	4*	11.8	0.221
		Yes		70	44.3		49	31		33	20.9	
	Sexual partner who injected drugs, lifetime											
		No		18	24	**<0.001**	9	12	**<0.001**	5	6.67	**<0.001**
		Yes		65	56.5		48	41.7		33	28.7	
	Had sexual partner who had been incarcerated, lifetime											
		No		12	26.7	**0.011**	6	13.3	**0.007**	4*	8.89	**0.044**
`		Yes		73	48		52	34.2		34	22.4	

*The chi-squared test was used to assess differences in proportion except when an expected value was less than five, in which

case Fisher’s Exact test was used (indicated by “*”).

Respondents who answered "Don’t Know" or refused to answer were removed from subsequent analyses.

1. Comparison refers to testing HIV positive versus testing HIV negative.

2. Comparison refers to testing HCV positive or self-reported history of HCV versus never testing positive for HCV.

3. Comparison refers to HIV/HCV co-infection by testing or history versus no HIV/HCV coinfection.

4. The p-value represents the middle category “neither agree nor disagrees” versus both agree or disagreeing with the statement receiving emotional support from family, using Fisher’s exact test.

### Multinomial analysis: Correlates of HIV, HCV, and HIV/HCV co-Infection compared to no infection

In multinomial analysis, independent correlates of HIV only, HCV only, and HIV/HCV co-infection compared to no infection are shown in Table 3. Ever injecting drugs was significantly associated with HIV infection (adjusted odds ratio [AOR] 3.52, 95% confidence interval [CI] 1.07–11.59), HCV infection alone (AOR 58.33, 95% CI 11.07–307.21), and HIV/HCV co-infection (AOR 31.21, 95% CI 8.14, 119.69). The factor with the highest magnitude of association with HIV infection was having emotional support from family (AOR 7.16, 95% CI 2.43–21.06) followed by neither agreeing nor disagreeing with having emotional support from family (AOR 6.82, 95% CI 1.67, 27.93). Receiving emotional support from family was also positively associated with HCV alone (AOR 8.35, 95% CI 1.78–39.05) and with HIV/HCV co-infection (AOR 5.54, 95% CI 1.59–19.33. Having a sexual partner who had ever injected drugs was associated with HIV infection alone (AOR 3.31, 95% CI 1.30–8.40). Cost not being a barrier to healthcare was associated with HIV/HCV co-infection (AOR 7.51, 95% CI 1.05–53.88). Increasing age was associated with HCV infection (AOR per year 1.09, 95% CI, 1.03–1.16), and HIV/HCV co-infection (AOR per year 1.10, 95% CI 1.04–1.16).

**Table 3:** Multinomial logistic regression associations with HIV, HCV, and co-infection, trans women in San Francisco, 2018–2020.

	Model I:			Model II:			Model III:		
	HIV infection only (n = 47) vs no infection (n = 96)			HCV infection only (n = 20) vs no infection (n = 96)			HIV/HCV co-infection (n = 38) vs no infection (n = 96)		
	Adjusted odds ratio	95% confidence interval	p-value	Adjusted odds ratio	95% confidence interval	p-value	Adjusted odds ratio	95% confidence interval	p-value
Associated factor									
Injection drug use, ever	3.52	1.07, 11.59	0.038	58.33	11.07, 307.21	<0.001	31.21	8.14, 119.69	<0.001
Sexual partner who injected drugs, lifetime	3.31	1.30, 8.40	0.012	--	--	--	2.96	0.80, 10.97	0.104
Did not lack care due to cost, last 12 months	--	--	--	--	--	--	7.51	1.05, 53.88	0.045
Receives emotional support from family	7.16	2.43, 21.06	<0.001	8.35	1.78, 39.05	0.007	5.54	1.59, 19.33	0.007
Neither agrees nor disagrees receives emotional support from family	6.82	1.67, 27.93	0.008	--	--	--	0.15	0.01, 2.17	0.163
Black/African American, non-Hispanic/Latina/e/x	--	--	--	3.06	0.62, 15.11	0.170	--	--	--
Ever incarcerated	--	--	--	--	--	--	3.92	0.93, 16.41	0.062
Age, per year	--	--	--	1.09	1.03, 1.16	0.006	1.10	1.04, 1.16	0.001

### HCV care cascade

**Fig 2:**
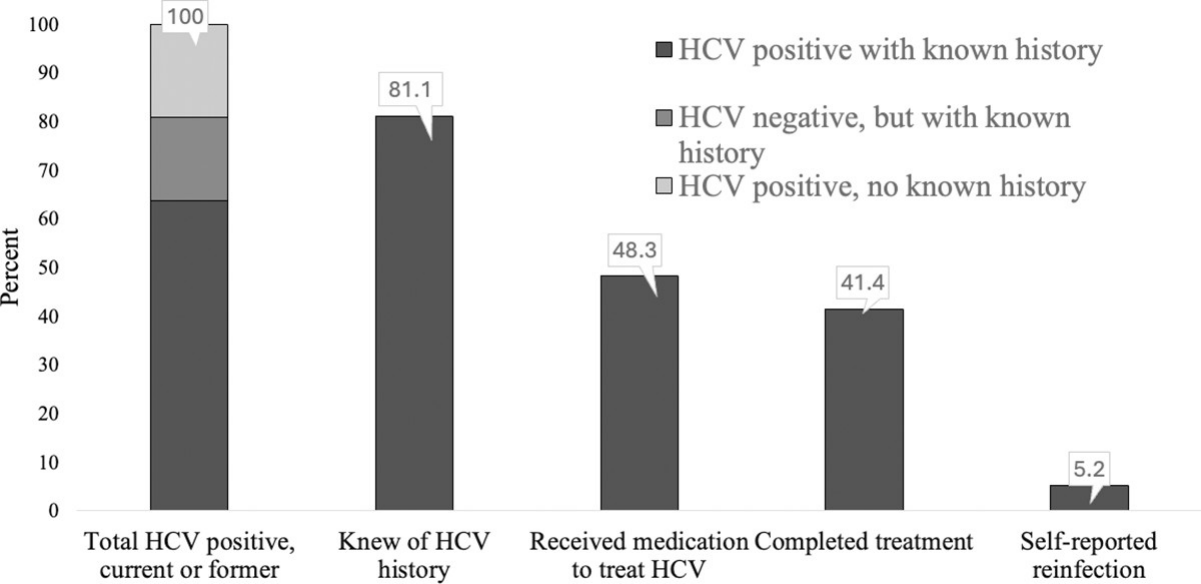
HCV care cascade. Fig 2 shows the cascade of engagement in HCV care: 81.1% of trans women with HCV were previously diagnosed, of whom 59.6% (28/47) received treatment, of whom 85.7% (24/28) completed treatment and 12.5% (3/24) reported reinfection.

Of the 201 trans women in our sample, 180 (89.6%) had ever been tested for HCV. Eleven trans women tested HCV positive for the first time in the current study, 37 tested HCV positive in the current study and also reported a history of known HCV infection, while 10 trans women reported a history of HCV infection but tested negative in the current study (Fig 2). Therefore, a total of 58 (28.9%) trans women had a current positive antibody test or a history of HCV infection. Forty-seven of the 58 trans women with evidence of HCV infection (81.0%) reported a past positive test, 26 of whom (26/47, 55.3%) reported a past positive viral RNA HCV test, and 28 of whom (28/47, 59.6%) received medication to treat HCV, with 24 (24/28, 85.7%) completing treatment. Six trans women of the 47 with a history of HCV diagnosis (6/47, 12.8%) were told by a provider their HCV infection spontaneously cleared, while 3 (3/47, 6.4%) were told they had re-infection after cure.

## Discussion

Our study found nearly half of trans women had evidence of current HIV infection, and one in three had evidence of past HCV infection. One in five trans women had evidence of both infections. These findings demonstrate an interaction between these two infections as the joint prevalence (i.e., co-infection) is 50% greater than would be expected by chance given their separate probabilities in the population. A systematic review of transgender populations found the prevalence of HIV ranged from 0% to 49.6%, and from 3.2% to 15.7% for HCV [27]. Notably, HCV prevalence in our study is above the range found in this review, documenting this current study population as the most severely affected among trans women, perhaps worldwide. For example, our HIV/HCV co-infection level of 18.9% is substantially higher than comparably measured data of trans persons in Pakistan at 5.6% (n = 87/1562) [28]. In a survey of female sex workers in India, HIV/HCV co-infection was substantially lower at 1.2% (3/250) HIV/HCV co-infection, despite a comparably high HIV prevalence of 42% (105/250). To our knowledge, few studies have looked at the prevalence of HIV/HCV co-infection in the total population of trans women. Other studies in the US, such as the Centers for AIDS Research Network of Integrated Clinical Systems (CNICS), focus on co-infection rates among trans women living with HIV and found that 23.6% of trans women living with HIV were co-infected with HCV [29]. A study in Italy found that in a sample of those undergoing sex reassignment surgery, 57.1% of HCV-positive trans women were also HIV-positive [30].

Multinomial modeling of the risk factors for HIV and HCV infections and HIV/HCV co-infection indicates that injection drug use is the most common shared mode of transmission. In bivariate analysis, having a sexual partner who was ever incarcerated and a partner who ever injected drugs were associated with HIV, HCV, and HIV/HCV co-infection. However, after controlling for trans women’s own injection behavior and incarceration history, sexual partners’ injection was only significantly associated with HIV infection alone. There has been debate in the literature on the sexual contribution to HCV transmission [31]. Transmission through sexual exposure may be less efficient compared with transmission through percutaneous exposures, although sex with an infected partner has been identified in some studies as a risk factor for HCV transmission [10, 11, 32]. Populations of men who have sex with men have been found with a high HCV burden among those who are also HIV-infected: for example, co-infection was 6.4% in a recent review [27], whereas HCV prevalence was as high as 82.4% in populations of people who inject drugs [11]. Our data contribute evidence that the synergy between HIV and HCV transmission may lie most strongly in injection-related behaviors.

Our findings and the literature also point to socio-structural factors that may be common to both infections. In the US, there are demographic disparities with HIV infection, with Black/African-Americans consistently showing a higher prevalence [32–34]. Our study did not confirm this disparity for Black/African American trans women [6, 20] in multinomial analysis when HCV infection is not included in the referent group. A history of unstable housing, substance abuse, being a sex worker, and incarceration have been associated with HIV, HCV independently, or co-infection [32, 35–41]. Risk may be amplified for people who inject drugs with any of the aforementioned risk factors [42–44], or overshadowed after controlling for injection drug use. Implementing intervention strategies for people who inject drugs has been shown to reduce HCV incidence, especially when combining multiple approaches (e.g., reduction of injection frequency, adoption of safer injection practices through sterile syringes and drug-preparation equipment, and behavior-change counseling) [42, 45]. Globally, roughly three in five people who inject drugs have a lifetime history of incarceration, due to factors such as the criminalization of drug use [39]. Incarceration can be a high-risk environment perpetuating transmission of HCV and HIV, especially when there is less access to sterile equipment and integrated healthcare [46]. Interventions reducing incarceration itself and more robust screening and treatment efforts for persons in prisons may be effective in reducing HIV and HCV transmission. Interventions designed for those with intersectional identities (e.g., sexual-gender-racial/ethnic minorities) may reach those at highest risk and greatest need most effectively [20].

The roles of access to healthcare and familial support on HIV, HCV, and HIV/HCV co-infection were complex in our data. Cost not being a barrier to healthcare was positively associated with HIV/HCV co-infection. Receiving emotional support from family was associated with all three: HIV-positive status, HCV infection, and HIV/HCV co-infection. The interpretation of this finding is not clear. A possible explanation may be when trans women are engaged long-term in HIV care, they tend to have more support, including family support in addition to healthcare coverage. Familial support has been associated with better mental health, higher levels of condom use, and less unprotected sex among trans women [47, 48]. However, indicating neither agreeing nor disagreeing with receiving emotional support from family was positively associated with HIV but not associated with HCV only or HIV/HCV co-infection. Compared to HIV-negative trans women, fewer HIV-positive trans women strongly disagreed they received emotional support from family. It is possible that support networks for trans women with HCV and HIV are both strong, but different. Both HIV and chronic HCV require continued care. Unlike HIV, HCV is curable and does not necessarily need the same lifelong support structures and treatment as HIV. Additionally, HIV may be perceived as a more life-threatening infection than HCV and therefore may garner more family support. Direct-acting antiviral treatment for HCV, developed in 2011, were a major advancement in HCV treatment that achieved a 90% or higher success rate of virological response [49]. The role stigma plays in HIV and HCV treatment and infection may also differ. In a qualitative study of HIV/HCV co-infected patients, the stigma associated with HIV and HCV was examined [50]. Most participants found both HCV and HIV to be stigmatizing, due to their associations with either injection drug use and/or risky sex behaviors [46]. However, co-infected individuals found HIV to be more stigmatizing due to factors such as HIV being perceived as more deadly, societal attitudes toward people associated with HIV, with public ignorance about HCV. Some respondents regarded HIV and HCV to be equally stigmatizing due to their association with injection drug use [50]. Therefore, interventions may be needed to increase familial support among trans women, including the development of chosen family support. Overall, the role of emotional support merits closer examination considering the high rates of HIV, HCV and HIV/HCV co-infection.

Our study also provides data on engagement in treatment for HIV and HCV among trans women. The HIV care cascade is encouraging, as trans women meet the global 90-90-90 targets set by the Joint United Nations Programme on HIV/AIDS (UNAIDS) (where 90% of people living with HIV are aware of their status, 90% of whom start ART treatment, of whom 90% are virally suppressed) [51]. These benchmarks were at 93-90-92 in our study [19]. Using contingent denominators, 92.9% of trans women with HIV were previously diagnosed, of whom 89.9% (71/79) were on ART, of whom 91.5% (65/71) reported being virally suppressed. The biggest fall-off for HIV appears to be starting ARV treatment after diagnosis. Recent initiatives for trans women in San Francisco (such as peer navigators, free gender-affirming surgery, and affordable housing) may have improved engagement in HIV care. The next step for HIV-positive trans women is to achieve 95-95-95 targets FastTrack Cities targets [52]. However, there are still major gaps with respect to engagement in HCV care. Only four-fifths of trans women with a history of HCV knew their status, of whom less than half had started treatment. Retention and completion of treatment were high among those who started, but there appears to be substantial re-infection. There needs to be an improvement at every step of the HCV cascade, especially increased HCV testing for trans women, and more initiatives to linkage to care if testing positive. More routine and periodic HCV testing is needed particularly for trans women who inject drugs, share needles/syringes, and are currently living with HIV.

We acknowledge the limitations of our data. Some measures in our cascades, including HCV viral load, prior test results, and completion of HCV treatment are based on self-reported data, which may over- or underestimate engagement in care. In determining HCV status, RNA testing was not available to determine current acute and chronic infections. We were also limited by the small number of HIV/HCV co-infected participants and small cell sizes for several comparisons. Finally, our data may not be fully representative of all trans women. It has been suggested that RDS may reach only the lower socio-economic strata of trans women [53], possibly due to the monetary incentive for participation. Even within the lower socioeconomic status groups, some segments of the population may not have participated. For example, two-thirds of trans women in our sample had a history of incarceration, with one in seven incarcerated in the last year, suggesting a substantial proportion of trans women would have been in detention at the time of the survey. External validity may also be limited as trans women in San Francisco may not be representative of other cities in the US. Unfortunately, the census data do not include transgender status that would permit assessing demographic differences between our sample and the larger trans women population.

In summary, our data provide positive and negative news for the HIV and HCV epidemics among the disproportionately affected population of trans women. HIV prevalence remains extremely high. This demonstrates a need for primary prevention of HIV to be strengthened. One potential avenue to strengthen primary prevention for HIV is through the uptake of PrEP, which had improved in San Francisco by the time of our survey. As of 2019, 94% of HIV-negative trans women in San Francisco were aware of PrEP, 65% had talked with a provider about PrEP, and 45% had used PrEP in the last 12 months [54]. Strong interventions are needed to improve HCV care and treatment, especially to test HCV-positive trans women and to connect HCV-positive trans women to treatment once they receive a diagnosis. Overall, more research on the effect of HIV/HCV co-infection for trans women is needed, especially concerning response to treatment and re-infection, considering HIV and HCV’s interactions and concurrent risk factors. Future research needs to probe deeper into HIV and HCV’s biological and immunological interactions, and how their treatment interacts with feminizing hormone replacement therapy. Meanwhile, scale-up of health and social welfare interventions for trans women who inject drugs may have the greatest effect on reducing these dual epidemics.
